# A combined biomarker panel shows improved sensitivity for the early detection of ovarian cancer allowing the identification of the most aggressive type II tumours

**DOI:** 10.1038/bjc.2017.199

**Published:** 2017-06-29

**Authors:** Matthew R Russell, Ciaren Graham, Alfonsina D'Amato, Aleksandra Gentry-Maharaj, Andy Ryan, Jatinderpal K Kalsi, Carol Ainley, Anthony D Whetton, Usha Menon, Ian Jacobs, Robert L J Graham

**Affiliations:** 1 Stoller Biomarker Discovery Centre and Pathology Node, Division of Molecular and Clinical Cancer Sciences, Faculty of Biology, Medicine and Health, University of Manchester, Oxford Road Manchester, Manchester, UK; 2 Centre for Biomedicine, School of Healthcare Science, Manchester Metropolitan University, Manchester, UK; 3 Gynaecological Cancer Research Centre, Women’s Cancer, Institute for Women’s Health, University College London, London, UK; 4 University of New South Wales, Kensington, NSW, Australia

**Keywords:** ovarian cancer, early detection, biomarkers

## Abstract

**Background:**

There is an urgent need for biomarkers for the early detection of ovarian cancer (OC). The purpose of this study was to assess whether changes in serum levels of lecithin-cholesterol acyltransferase (LCAT), sex hormone-binding globulin (SHBG), glucose-regulated protein, 78 kDa (GRP78), calprotectin and insulin-like growth factor-binding protein 2 (IGFBP2) are observed before clinical presentation and to assess the performance of these markers alone and in combination with CA125 for early detection.

**Methods:**

This nested case–control study used samples from the United Kingdom Collaborative Trial of Ovarian Cancer Screening trial. The sample set consisted of 482 serum samples from 49 OC subjects and 31 controls, with serial samples spanning up to 7 years pre-diagnosis. The set was divided into the following: (I) a discovery set, which included all women with only two samples from each woman, the first at<14 months and the second at >32 months to diagnosis; and (ii) a corroboration set, which included all the serial samples from the same women spanning the 7-year period. Lecithin-cholesterol acyltransferase, SHBG, GRP78, calprotectin and IGFBP2 were measured using ELISA. The performance of the markers to detect cancers pre-diagnosis was assessed.

**Results:**

A combined threshold model IGFBP2 >78.5 ng ml^−1^ : LCAT <8.831 *μ*g ml^−1^ : CA125 >35 U ml^−1^ outperformed CA125 alone for the earlier detection of OC. The threshold model was able to identify the most aggressive Type II cancers. In addition, it increased the lead time by 5–6 months and identified 26% of Type I subjects and 13% of Type II subjects that were not identified by CA125 alone.

**Conclusions:**

Combined biomarker panels (IGFBP2, LCAT and CA125) outperformed CA125 up to 3 years pre-diagnosis, identifying cancers missed by CA125, providing increased diagnostic lead times for Type I and Type II OC. The model identified more aggressive Type II cancers, with women crossing the threshold dying earlier, indicating that these markers can improve on the sensitivity of CA125 alone for the early detection of OC.

Ovarian cancer (OC) has the highest mortality rate of all the gynaecological cancers with an estimated 15 500 deaths in the United States in 2012 alone (Siegel *et al*, 2012). This is due to its typically late diagnosis, with 5-year survival rates of 5% in those diagnosed at Stage IV. If diagnosed early, at stage I, the 5-year survival rate rises to >90% (CRUK, 2014). There is an unmet need for diagnostic tests that enable earlier diagnosis, which would increase survival.

Ovarian cancer can be classified into Type I (more indolent tumours lacking mutations in TP53) and Type II (aggressive cancers displaying TP53 mutations in >80% of cases), which account for most of the OC mortality) (Kurman and Shih, 2010). Serum CA125 is the only biomarker currently used to triage and monitor patients (Jacobs and Bast, 1989), with a threshold of 35 U ml^−1^ triggering referral to a gynaecological oncologist (Cramer *et al*, 2011). However, CA125 elevation is not unique to OC, as it is also raised during menstruation, pregnancy and endometriosis, and it is only elevated in ∼50% of early stage cancers (Jacobs and Bast, 1989). Extended combinations of biomarkers should offer higher discriminatory power. A putative panel of biomarkers, identified at OC diagnosis, from the Prostate, Lung, Colorectal and Ovarian cancer study (Cramer *et al*, 2011) proved unable to detect OC in pre-clinical samples (Zhu *et al*, 2011), suggesting putative biomarkers were elevated at clinical presentation but not present earlier in the natural history of the disease.

The prospectively collected serum sample set generated during the United Kingdom Collaborative Trial of Ovarian Cancer Screening (UKCTOCS) (Menon *et al*, 2008; Menon *et al*, 2009a; Menon *et al*, 2015) offers the possibility of discovering biomarkers in pre-clinical serum samples. A previous study by the authors’ using isobaric tags (iTRAQ) identified 90 proteins differentially expressed between OC cases and controls. A second targeted mass spectrometry analysis of twenty of these putative biomarkers led to the successful validation of Protein Z as a potential early detection biomarker for OC from the UKCTOCS sample set (Russell *et al*, 2016). Here, a further five putative biomarkers identified in this previous work, lecithin-cholesterol acyltransferase (LCAT), insulin-like growth factor-binding protein 2 (IGFBP2), sex hormone-binding globulin (SHBG), glucose-regulated protein, 78 kDa (GRP78) and calprotectin (uniprot accession numbers P04180, P18065, P04278, P11021 and complexed P05109 and P06702), were investigated for their utility in OC screening. Insulin-like growth factor-binding protein 2 and SHBG are both hormone-binding proteins that have previously been implicated in OC (Flyvbjerg *et al*, 1997; Baron-Hay *et al*, 2004; Nolen and Lokshin, 2012; Gharwan *et al*, 2015). Lecithin-cholesterol acyltransferase has previously been shown to be downregulated in breast cancer at diagnosis (Hilal Kiziltunc and Askin, 2013). Calprotectin is an antibacterial cytosolic protein found most abundantly in neutrophils and upregulation of serum levels occur during inflammation (Striz and Trebichavsky, 2004); it has previously been shown to be upregulated in plasma of women with OC (Odegaard *et al*, 2008). Glucose-regulated protein, 78 kDa is a glucose-regulated protein, which controls protective mechanisms during stress; it has been shown to be upregulated in malignant breast lesions (Fernandez *et al*, 2000) and via association with STMN1 to promote metastasis in such tumours (Kuang *et al*, 2016).

We therefore sought to investigate the performance of this panel in combination with CA125 for early diagnosis of OC and their ability to identify the most aggressive subtypes in a nested case–control study within UKCTOCS.

## Materials and methods

### Serum samples

United Kingdom Collaborative Trial of Ovarian Cancer Screening (International Standard Randomised Controlled Trial, number ISRCTN22488978; ClinicalTrials.gov NCT00058032) is a randomised controlled trial of OC screening in the general population, approved by the UK North West Multicentre Research Ethics Committees (North West MREC 00/8/34). Trial design, including eligibility criteria and details of recruitment has been described in detail elsewhere (Menon *et al*, 2008; Menon *et al*, 2009a; Menon *et al*, 2015). All women provided written informed consent for use of their samples in secondary studies. The current biomarker discovery study was approved by the Joint UCL/UCLH Committees on the Ethics of Human Research (Committee A) (Reference Number 05/Q0505/57). Full details of sample acquisition, transport and storage and CA125 quantification in the sample set have been published previously (Menon *et al*, 2009a).

The serum set investigated here comprised eighty women; 49 women with OC, 30 Type II and 19 Type I (of which 10 were borderline and as with previous studies were grouped with Type I for analysis (Shih and Kurman, 2004; Wu *et al*, 2013; Russell *et al*, 2016); 31 control samples were selected from women in the study, who had no diagnosis of a cancer during follow-up and were matched by age, collection centre and collection date to the Type II samples. This set contained all of the invasive OC samples available that had serial samples spanning less than 14 months to diagnosis right through to greater than 32 months and up to 84 months to diagnosis. Multiple serial samples were available from these 80 women so the full sample set comprised 482 individual samples spanning up to seven years prior to diagnosis (time to diagnosis, tDx).

The set was divided into the following: (i) a discovery set, which comprised two samples per woman, one at <14 months and the other at >32 months tDx; and (ii) a corroboration set, which comprised the additional serial samples from the same women spanning a 7-year period tDx.

### Serum measurements

ELISA assays for IGFBP2 and LCAT (Cloud Clone Corp., Wuhan, Hubei, China), SHBG (R&D Systems, Abingdon, UK), GRP78 (Enzo Life Sciences, Exeter, UK) and calprotectin (Biolegend, San Diego, CA, USA) were performed in duplicate using commercial kits following manufacturers’ instructions.

The mean coefficients of variance for duplicate analysis for each assay were as follows: IGFBP2: 8.1% LCAT: 8.4% SHBG: 7.4% GRP78: 3.1% and calprotectin: 4.5%. Serum CA125 levels were available as previously described (Menon *et al*, 2009a).

### Statistical analysis

All analysis was performed using the R environment for statistical analysis. The Mann–Whitney test was used to assess significance of differences. The Fisher’s exact test was used to assess significance of categorical data. Correlations were assessed using Pearson’s product moment. Logic-rule-based threshold models were constructed to investigate the biomarkers discriminatory power (O'Brien *et al*, 2015). Differences in sensitivity between models were assessed with McNemar’s test. The areas under the curve of receiver operating characteristics curves were calculated for threshold models. Significance of lead time improvement was calculated with a paired *t*-test. All tests were two tailed and those with *P*-values <0.05 were considered statistically significant.

## Results

### Study set characteristics

The baseline characteristics of the study participants and tumour characteristics have previously been reported (Russell *et al*, 2016) and are provided as Supplementary Data (Supplementary Tables S1 and S2). Subjects’ BMI, current HRT and oral contraceptive pill use were recorded at recruitment, and HRT use and smoking recorded in the first follow-up questionnaires sent to all participants 3–5 years post randomisation. None of these factors correlated with OC diagnosis.

### Serum levels of IGFBP2, SHBG, LCAT, GRP78, calprotectin and CA125 in the discovery set

**Figure 1:**
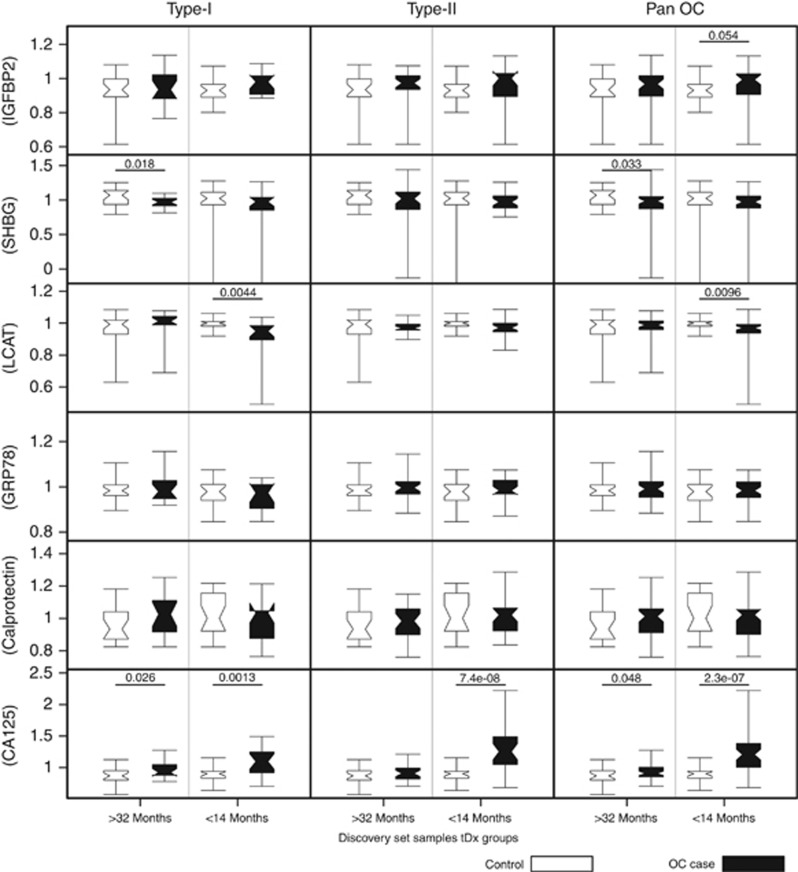
**Box plot showing putative biomarker expression in the discovery sample set at >32 months tDx and <14 months tDx.** The whisker limits represent the 5th and 95th percentiles; the box limits represent interquartile range; the closest point in the notches (><) of the box plot represents the median and the span from the bottom to the top of the notch is 95% confidence interval (for significant values between cases and controls it can be seen that these do not overlap). Significant *P-*values are indicated on the plot. (For this initial triage the value for IGFBP2 is shown as it is close to the cutoff value).

In the discovery set, IGFBP2, SHBG, LCAT, GRP78 and calprotectin were quantified using commercial ELISA kits, whereas CA125 levels were available from the main UKCTOCS trial database (Menon *et al*, 2009a). Protein expression was log transformed and scaled to unit variance allowing direct comparison between markers (Figure 1). The expression of these markers at <14 and >32 months to diagnosis were analysed separately for Type I and Type II OC cases (to ascertain whether the proteins were potential subtype-specific early detection markers). The results were then combined and analysed to ascertain their potential as pan OC early detection biomarkers.

Insulin-like growth factor-binding protein 2 showed no significant change in the Type I or Type II individual analysis but did show upregulation in the pan OC analysis <14 months to diagnosis (*P*=0.054). Sex hormone-binding globulin serum levels were significantly downregulated in the Type-I (*P*=0.018) and the pan OC (*P*=0.033) >32 months to diagnosis. Although LCAT showed significant downregulation in Type-I (*P*=0.0044) and pan OC (*P*=0.0096) <14 months to diagnosis. Neither GRP78 nor calprotectin showed significant regulation compared with controls. CA125 showed significant upregulation in serum for Type II (*P*=7.4 × 10^−8^) cases at <14 months to diagnosis. In addition, it showed significant upregulation for both Type I <14 months (*P*=0.0013) and >32 months (*P*=0.026), and for pan OC <14 months (*P*=2.3 × 10^−7^) and >32 months (*P*=0.048) in these samples.

### Correlation of biomarker expression with epidemiological factors

Correlation of biomarker expression with epidemiological factors was investigated within the discovery set. BMI and contraceptive pill use (‘Have you ever taken the oral contraceptive pill?—yes or no’) assessed at recruitment and age-at-sample were significantly correlated only with SHBG (*P*=0.009, *P*=0.034 and *P*=0.043, respectively). HRT use at recruitment (‘Are you currently on hormone replacement therapy (HRT)?—yes or no’) was correlated with IGFBP2 (*P*=0.01) and SHBG (*P*=0.03) expression but HRT use at follow up (‘Are you currently taking HRT?’—yes or no) showed no correlations with expression. Contraceptive pill use at recruitment was correlated with SHBG (*P*=0.034). Smoking assessed at follow up (‘Have you ever been a smoker?—yes or no’) was not correlated with any biomarker expression.

### Combined analysis of discovery and corroboration set

IGBP2, LCAT and SHBG were taken forward for further analysis and were quantified in the additional samples of the corroboration set. Protein expression was log transformed and scaled to unit variance for comparison. As with the discovery set protein expression was analysed separately for Type I and Type II OC cases and the results then combined to investigate their potential as pan OC early detection biomarkers.

**Figure 2:**
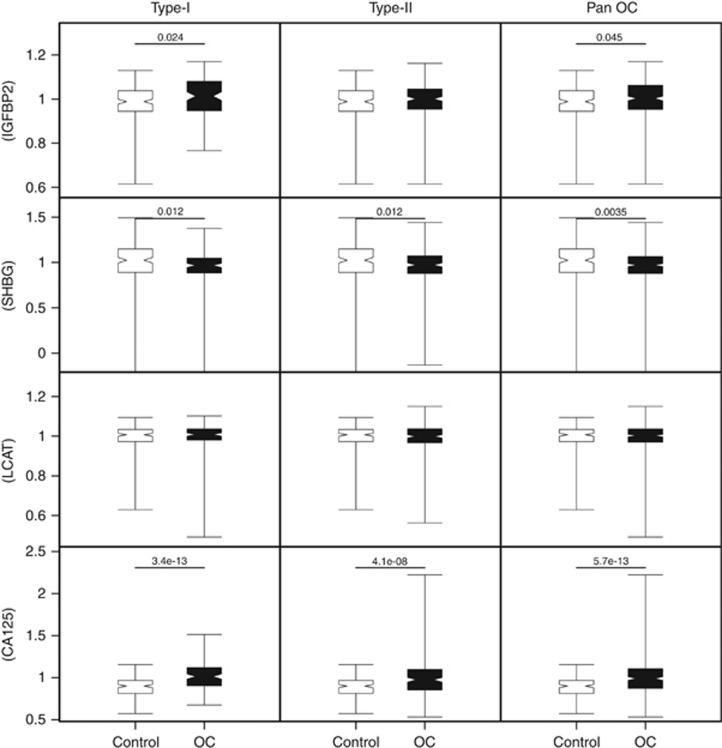
**Comparison of putative biomarker expression in pre-diagnosis sets with no division by tDx.** The whisker limits represent the 5th and 95th percentiles; the box limits represent interquartile range; the closest point in the notches (><) of the box plot represents the median and the span from the bottom to the top of the notch is 95% confidence interval (for significant values between cases and controls it can be seen that these do not overlap). Significant *P-*values are indicated on the plot.

**Figure 3:**
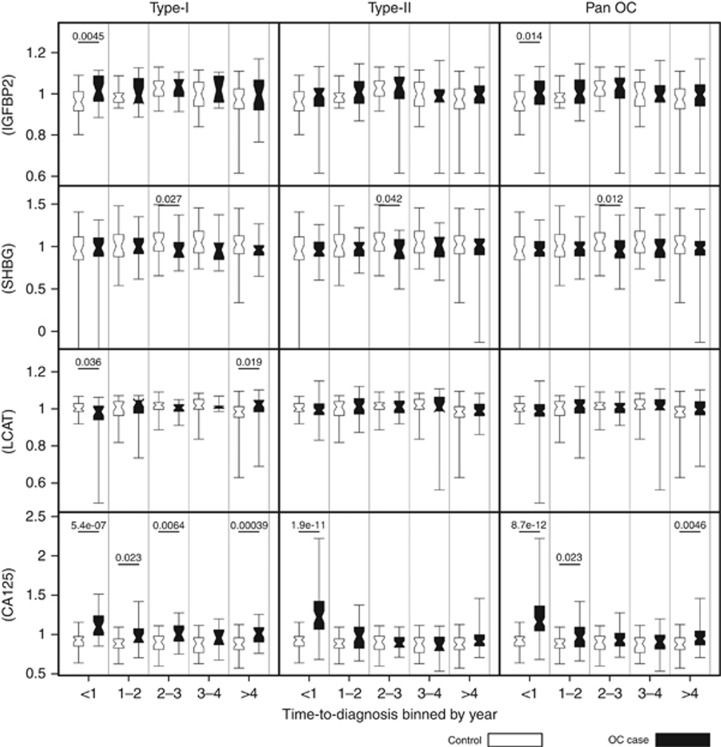
**Comparison of putative biomarker expression in pre-diagnosis sets, divided into yearly intervals.** The whisker limits represent the 5th and 95th percentiles; the box limits represent interquartile range; the closest point in the notches (><) of the box plot represents the median and the span from the bottom to the top of the notch is 95% confidence interval (for significant values between cases and controls it can be seen that these do not overlap). Significant *P-*values are indicated on the plot.

There are two potential applications for biomarker panels in the early detection of OC. One is their potential use as aids in clinical ‘triage’ for symptomatic/high-risk patients. Analysis of the biomarker levels from a single blood sample, with no temporal information, at primary care centres could be used to ascertain the risk of OC. This is investigated in Figure 2, where the levels of the potential biomarkers are compared to control levels. The second application would be as part of a ‘screening’ programme. Here, the levels of the potential markers would be followed temporally to determine if they indicated early disease initiation and progression, this is outlined in Figure 3.

### Insulin-like growth factor-binding protein 2, SHBG and CA125 levels display differential expression for OC triage

The levels of IGBP2, LCAT, SHBG and CA125 were compared directly with those of the control population in all samples (Figure 2). Insulin-like growth factor-binding protein 2 was significantly upregulated in Type-I (*P*=0.024) and in the pan OC (*P*=0.045). SHBG was significantly downregulated in Type-I (*P*=0.012) and Type-II (*P*=0.012), and in the pan OC (*P*=0.0035). Lecithin-cholesterol acyltransferase displayed no differential expression at this stage of the analysis. CA125 was significantly upregulated in Type-I (*P*=3.4 × 10^−13^) and Type-II (*P*=4.1 × 10^−8^), and in the pan OC (*P*=5.7 × 10^−13^).

### Insulin-like growth factor-binding protein 2, SHBG, LCAT and CA125 display differential expression pre-diagnosis for screening

The levels of all of the biomarkers were compared directly with those of the control population at yearly intervals, from diagnosis up to 4 years pre-diagnosis after which all time points >4 years tDx were grouped together (Figure 3). Insulin-like growth factor-binding protein 2 was significantly upregulated: in Type-I samples at <1 year tDx (*P*=0.0045) and for pan OC at <1 year tDx (*P*=0.014). Sex hormone-binding globulin was significantly downregulated in Type-I samples at 2–3 years tDx (*P*=0.027) and at the same time point in Type-II (*P*=0.042) and in pan OC (*P*=0.012). Lecithin-cholesterol acyltransferase showed significant downregulation in OC with respect to controls in Type-I samples <1 year tDx (*P*=0.036) and upregulation at >4 years tDx (*P*=0.019).

CA125 showed significant upregulation in Type I samples at <1 year (*P*=5.4 × 10^−7^), 1–2 (*P*=0.023), 2–3 (*P*=0.0064) and >4 years tDx (*P*=0.00039). In Type II cases it displayed significant upregulation <1 year tDx, whereas in the pan OC comparison it demonstrated upregulation <1 (*P*=8.7 × 10^−12^), 1–2 (*P*=0.023) and >4 years tDx (*P*=0.0046).

#### Triage

Sensitivities for detection of OC using the putative biomarkers were assessed by applying selected cutoffs and an ‘OR’ rule (O'Brien *et al*, 2015) for the expression of each biomarker alone and in combination with each other and CA125 (Table 1), improvements in sensitivity have been highlighted in grey.

### Threshold models demonstrate improved sensitivity for OC

The selected thresholds were LCAT: <8.831 *μ*g ml^−1^; SHBG: <16.1 nmol l^−1^; and IGFBP2: >78.5 ng ml^−1^ set to give a 5% false positive rate. The threshold for CA125 was taken as the level at which a woman would be referred to a gynaecological oncologist 35 U ml^−1^ (Cramer *et al*, 2011). With a small decrease in specificity, the combined panels all yielded dramatically increased sensitivity over CA125 alone for both Type I and Type II OC.

#### Screening

The putative biomarkers were again assessed, at yearly intervals, by applying selected cutoffs and an ‘OR’ rule (O'Brien *et al*, 2015) for the expression of each biomarker alone and in combination with each other and CA125 (Supplementary Table S3); statistically significant improvements in sensitivity are shown in orange.

### Individual models outperform CA125

Although individual threshold models (IGBFP2/SHBG/ LCAT) displayed increased sensitivity over CA125 alone for various time points for Type I, Type II and Pan OC, they were not statistically significant.

### Individual markers combined with CA125 models outperform CA125 alone

The IGBFP2 : CA125 threshold model outperformed CA125 (Supplementary Table S3) in Type I OC at >4 year tDx where its sensitivity was >5 times that of CA125; however, the specificity was slightly lower. For Type II patients it outperformed CA125 at 1–2 years tDx where sensitivity tripled. It also outperformed CA125 in terms of sensitivity at >4 years tDx, where its sensitivity quadrupled but with slightly lower specificity. In the pan OC analysis, it outperformed CA125 at <1 and at 1–2 years tDx. It also outperformed CA125 in terms of sensitivity at 2–3 and >4 tDx, but with a slight decrease in specificity.

The SHBG : CA125 model did not significantly outperform CA125 (Supplementary Table S3) in the Type I, Type II or pan OC analysis.

The LCAT : CA125 model only out performed CA125 for pan OC at <1 year tDx.

### Combination biomarker models outperform CA125 alone

**Figure 4:**
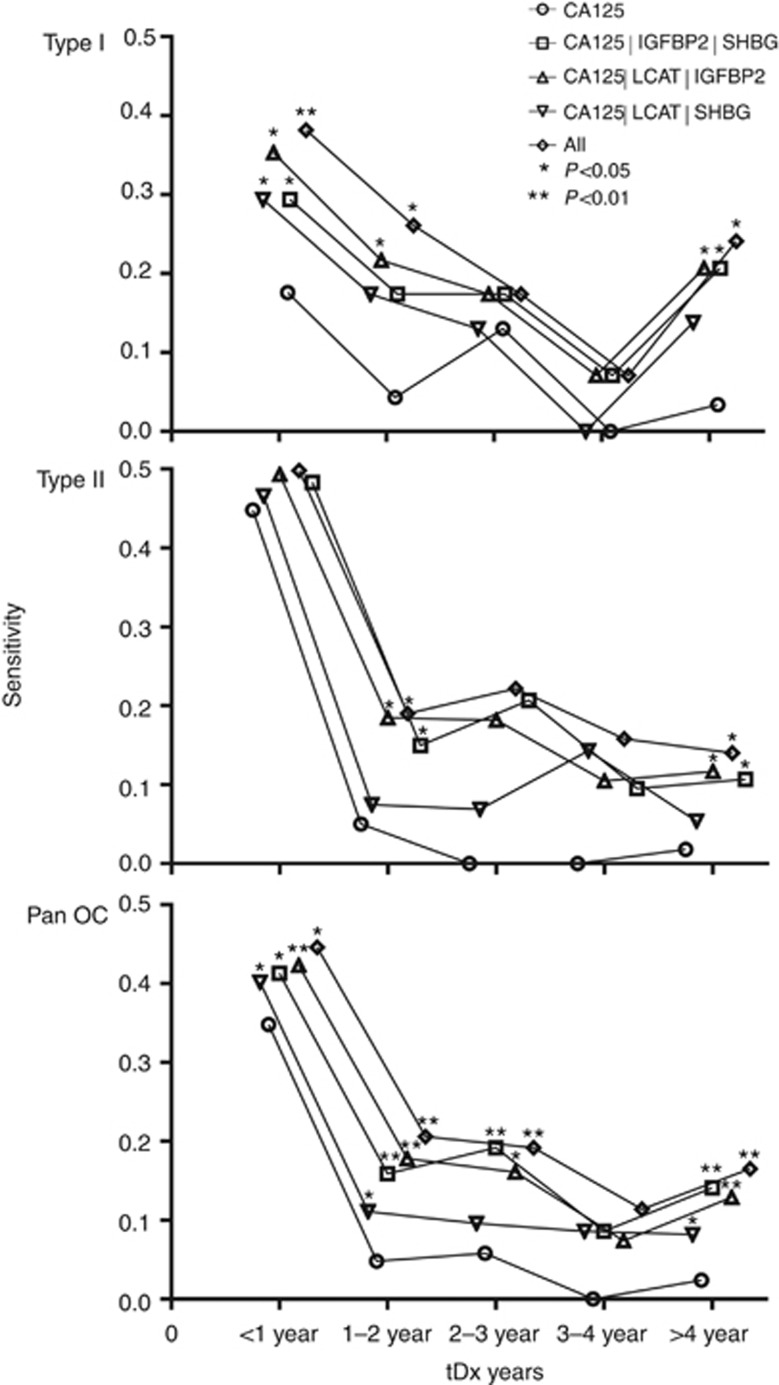
**Graph showing the increase in sensitivity, over time, of the combined threshold models *versus* CA125 alone for Type I OC, Type II OC and Pan OC.** Significant *P-*values are indicated on the plot.

Figure 4 shows the performance, in terms of sensitivity, of the multiple marker combinations in conjunction with CA125. The plots demonstrate an improvement in sensitivity for all the combination panels compared with CA125 alone for Type I, Type II and Pan OC.

The SHBG : IGFBP2 : CA125 model most strikingly significantly outperformed CA125 alone in Type I OC at <1 and >4years to diagnosis. Although for Type II it significantly outperformed CA125 at 1–2 and >4 years tDx. In the pan OC analysis, it also outperformed CA125 in terms of sensitivity at all times points, with only 3–4 years tDx not displaying significance.

The LCAT : SHBG : CA125 model significantly outperformed CA125, for Type I at <1 year tDx. For Type II patients, it outperformed CA125 at all time points, but with no statistical significance. In the pan OC analysis, it significantly outperformed CA125 at >4, 1–2 and <1 year to tDx.

The IGFBP2 : LCAT : CA125 model significantly outperformed CA125 in Type I OC at >4, 1–2 and <1 year tDx. For Type II patients, it significantly outperformed CA125 at >4 and 1–2 years to tDx. In the pan OC analysis, it also outperformed CA125 in terms of sensitivity at all times points, with only 3–4 years tDx not displaying significance.

The combination of all markers (LCAT : SHBG : IGFBP2 : CA125) model significantly outperformed CA125 (Figure 4) in Type I OC at >4, 1–2 and <1 years tDx. For Type II patients, it significantly outperformed CA125 at >4 and 1–2 years to tDx. In the pan OC analysis, it also outperformed CA125 in terms of sensitivity at all times points, with only 3–4 years tDx not displaying significance.

Although the all marker combination performs well, its specificity is lowered by the presence of SHBG (Supplementary Table S3). This means that the IGFBP2 : LCAT : CA125 model provided the best improvement in performance for the detection of OC over CA125 alone.

### Lead time estimation

For Type-I OC, 11 women were not detected by either elevated CA125 or the combined IGFBP2 : LCAT : CA125 model. Of the remaining eight subjects, five were detected earlier by this combined model, of which four were not detected by CA125 alone. For the combined model the mean lead time was 454 days tDx, whereas for CA125 alone it was 315 days tDx (*P*=0.032).

For Type-II OC, 13 women were not detected either by CA125 or combined IGFBP2 : LCAT : CA125 model. Of the remaining 17 women, 4 were detected by the combined model earlier than CA125 alone and 2 of these were not detected by the CA125 threshold at all. For this combined model, the mean lead time was 272 days tDx, whereas for CA125 alone the lead time was 165 days tDx. Combining all OC together, gave a mean lead time for CA125 alone of 213 days and the IGFBP2 : LCAT : CA125 model of 330 days (*P*=0.014), a difference of 107 days equating to a four month improvement over CA125 alone.

### Prognosis

**Figure 5:**
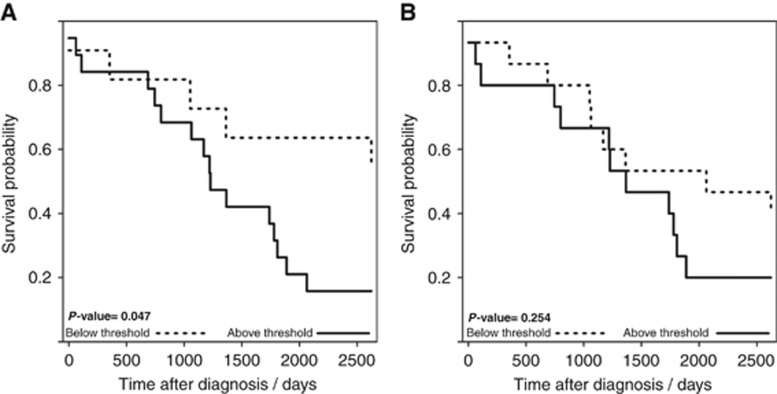
**Survival curves.** (**A**) for the combined threshold model (IGFBP2 : LCAT : CA125), (**B**) for CA125, using time to death post diagnosis. The dotted black line represents Type II patients who did not breach the threshold. The solid black line represents Type II patients with samples that breached the threshold.

An additional question that can be asked is does this IGFBP2 : LCAT : CA125 threshold model provide us with information on the aggressiveness of the OCs. This was investigated via Kaplan–Meier analysis, using the time from diagnosis to death. Plotting survival curves for the Type II patients that breach this threshold *versus* those that do not (Figure 5) confirmed a significant difference in survival curves; those patients that breach the IGFBP2 : LCAT : CA125 threshold model (Figure 5A) have a lower survival (*P*=0.047) than those that do not. This is directly attributable to the threshold model as Figure 5B shows that in survival curves based on CA125 alone there is no difference in survival between those who cross the CA125 threshold and those who do not (*P*=0.254) confirming IGFBP2 : LCAT : CA125 as a prognostic panel.

## Discussion

Despite intensive efforts over the past three decades to improve treatment (both surgery and chemotherapy) for the disease, there is still a poor outcome for women diagnosed with OC. In 2014, 7378 women in the United Kingdom were diagnosed with OC and there were 4128 deaths. The majority of OC cases are diagnosed at late stage, with a 5-year survival rate of <23% for Stage III and IV cancers (Nolen and Lokshin, 2012; Hüttenhain *et al*, 2012; CRUK, 2014). When detected, early prognosis is much better, with >90% of women diagnosed at Stage I surviving 5 years (CRUK, 2014). There is therefore a significant need to develop strategies, which can detect OC early. Serum biomarkers are attractive targets for early detection and indeed the serum marker CA125 has been widely used in screening trials for OC (Moore *et al*, 2009; Menon *et al*, 2009b; Cramer *et al*, 2011; Skates, 2012; Bristow *et al*, 2013; Drescher *et al*, 2013). However, CA125 has limitations of specificity for OC (Jacobs and Bast, 1989). Thus, there is a real need for the identification and development of biomarkers capable of improving on or complementing CA125 in order to enable the earlier detection of OC.

This is the first study to have investigated the expression of putative OC biomarkers IGFBP2, LCAT, SHBG, GRP78 and calprotectin in prospectively collected pre-clinical samples, enabling an unbiased assessment of how these markers alter during OC progression.

The most effective biomarker panel was a combination of IGFBP2 : LCAT : CA125. This panel identified 26% of Type I subjects and 13% of Type II subjects not identified by the CA125 threshold alone. This panel also displayed an increased lead time of 5–6 months for Type I and 3–4 months for Type II OC. It is important to note when considering the above lead time results that women enrolled on the UKCTOCS study were on average diagnosed between 1 and 2 years earlier than is typical in the unscreened population, giving potential lead times of 2–3 years offering a significant window for clinical intervention.

Kaplan–Meier plots also demonstrate that this threshold panel is discriminatory for more aggressive OC as Type II subjects who breach the threshold model have a lower survival rate than Type II patients who do not.

Following initial analysis of IGFBP2, LCAT, SHBG, GRP78 and calprotectin within our discovery set IGFBP2, LCAT and SHBG, were taken forward as potential markers for OC and further analysed within our corroboration set. Markers were analysed over a 7 years pre-diagnosis period and binned into yearly time windows (Figure 3). IGFBP2 displayed significant upregulation at <1 year tDx for the Type I and Pan OC analysis. SHBG displayed significant downregulation in Type I, Type II and Pan OC at 2–3 years to diagnosis, LCAT showed significant downregulation in Type I OC at <1 year tDx and significant upregulation at >4 years tDx. However, none of these markers outperformed CA125.

In order to ascertain whether the putative biomarkers could be constructed into a panel with CA125 that would improve on CA125 alone, for the early detection of OC, threshold models were investigated. These models were constructed for all members of the biomarker panel and combinations tested against CA125 alone. The combination of CA125 and IGBP2 improved the sensitivity for detection of OC for both Type I and Type II at >4 years and for Type II at 1–2 pre-diagnosis. The combination of CA125 and LCAT showed improvements against CA125 alone in the <1 year time range for Pan OC.

At the next level, CA125 was combined with two markers IGBP2 : SHBG, LCAT : SHBG and IGBP2 : LCAT. The most striking feature of the two panels containing SHBG is that it has a deleterious effect on the specificity of the models.

The most effective biomarker panel was IGFBP2 : LCAT : CA125; this panel outperformed CA125 in terms of sensitivity at nearly all time points measured for the Type I and Type II cancers, with at least a doubling in the sensitivity of the panel at 0–2 years’ pre-diagnosis for Type I OC and a greater than tripling in sensitivity for Type II at 1–2 and >4 years tDx. For Type I OC, 11 women were not detected by elevated CA125 or the combined IGFBP2 : LCAT : CA125 threshold. Of the remaining eight subjects, five were detected earlier by the combined threshold, of which four were not detected by CA125 alone. For the combined threshold, the mean lead time was 454 days tDx, whereas for CA125 alone it was 315 days tDx (*P*=0.032).

For Type II OC, 13 women were not detected either by CA125 or combined IGFBP2 : LCAT : CA125 thresholds. Of the remaining 17 women, 4 were detected by the combined threshold earlier than CA125 alone; of these, 2 were not detected by the CA125 threshold at all. For the combined threshold model the mean lead time was 272 days tDx, whereas for CA125 alone the lead time was 165 days tDx. Combining all OC together gave a mean lead time for CA125 alone of 213 days and the combined model of 330 days (*P*=0.014), a difference of 107 days equating to a 4-month improvement over CA125 alone.

This is the first study to have investigated the expression of putative OC biomarkers IGFBP2, LCAT and SHBG in prospectively collected pre-clinical samples. Treated as single markers, these proteins offered low sensitivities, but in a combined threshold model they were able to correctly identify OC in samples that did not breach the CA125 threshold, improving on the sensitivity of CA125 alone and identifying cases it missed. In addition, they provide an increased lead time of several months in the detection of OC over CA125 alone and importantly identified the more aggressive Type II cancers. Before their utility in a clinical setting can be assessed, these panels will need to be further validated in larger cohorts. However, the threshold models generated within this study demonstrate the potential of these biomarkers in improving the sensitivity and detection of OC as part of a panel incorporating CA125.

## Figures and Tables

**Table 1:**
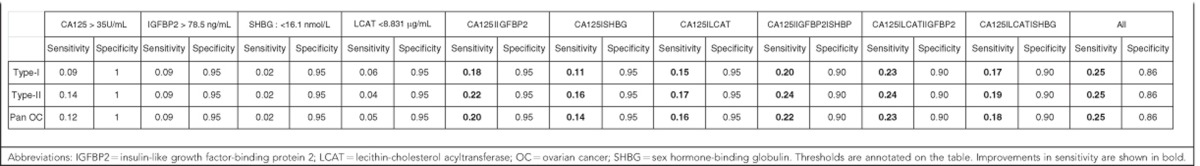
Performance of threshold models for samples, with no temporal information, for each of the putative biomarkers individually and in combination
