# Supplementary material for: A combined biomarker panel shows improved sensitivity for the early detection of ovarian cancer allowing the identification of the most aggressive type II tumours
Source: Br J Cancer. 2017 Jun 29;117(5):666–74. doi: 10.1038/bjc.2017.199 (PMC5572165; doi:10.1038/bjc.2017.199)
Supplement: Supplementary Table legends [file bjc2017199x1.docx]

**Supplemental legends:**

**Table S1:** Characteristics of the primary ovarian cancers in this study.

**Table S2:** Baseline characteristics of UKCTOCS participants used within this study.

**Table S3:** Performance of threshold models for samples grouped by time to diagnosis for each of the putative biomarkers individually and in combination. Thresholds are annotated on the table. The ROC-AUC summarises both sensitivity and specificity and enables ready comparison of any pair of model-time bin pairs. Improvements in sensitivity are shown in orange significant differences are highlighted in green.
