# Supplementary material for: A combined biomarker panel shows improved sensitivity for the early detection of ovarian cancer allowing the identification of the most aggressive type II tumours
Source: Br J Cancer. 2017 Jun 29;117(5):666–74. doi: 10.1038/bjc.2017.199 (PMC5572165; doi:10.1038/bjc.2017.199)
Supplement: Supplementary Table S1 [file bjc2017199x2.docx]

**Table S1:**

|  | **No.** | | | |
| --- | --- | --- | --- | --- |
| **Cancer type** | **Overall** | **Stage I** | **Stage II** | **Stage III** |
| **Type I** | **19** | **14** | **1** | **4** |
| **Borderline** | **10** | **10** | **0** | **0** |
| Serous | 6 | 6 | 0 | 0 |
| Mucinous | 2 | 2 | 0 | 0 |
| Endometrioid | 2 | 2 | 0 | 0 |
| **Invasive** | **9** | **4** | **1** | **4** |
| Low grade endometrioid | 5 | 3 | 1 | 1 |
| Clear cell | 3 | 0 | 0 | 3 |
| Adenocarcinoma | 1 | 1 | 0 | 0 |
| **Type II** | **30** | **7** | **8** | **15** |
| High grade serous | 23 | 5 | 6 | 12 |
| High grade endometrioid | 3 | 1 | 1 | 1 |
| Carcinosarcoma | 1 | 0 | 0 | 1 |
| Adenocarcinoma | 3 | 1 | 1 | 1 |
