# Supplementary material for: A combined biomarker panel shows improved sensitivity for the early detection of ovarian cancer allowing the identification of the most aggressive type II tumours
Source: Br J Cancer. 2017 Jun 29;117(5):666–74. doi: 10.1038/bjc.2017.199 (PMC5572165; doi:10.1038/bjc.2017.199)
Supplement: Supplementary Table S2 [file bjc2017199x3.docx]

| **Table S2:** | | | | | | | | | |
| --- | --- | --- | --- | --- | --- | --- | --- | --- | --- |
|  | **Median (25^th^ - 75^th^ centiles)** | | | | | | | |  |
|  | **Control** | | **Ovarian cancer** | | | | | |  |
|  |  |  | **Overall** | | **Type I** | | **Type II** | |  |
|  | **n=31** | | **n=49** | | **n=19** | | **n=30** | |  |
| **Age (years) at randomisation** | 60.8 (58.4-65.8) | | 62.8 (58.7-67.3) | | 64.2 (58.9-69.9) | | 61.1 (58.7-65.5) | |  |
| **Years since last period at randomisation** | 12.6 (6.6-18.2) | | 11.4 (5.7-18.2) | | 15.2 (8.1-22.6) | | 10.7 (4.0-16.1) | |  |
| **Duration of HRT use in those who were on HRT at randomisation (yrs)** | 6.9 (5.8-11.7) | | 9.7 (4.8-13.0) | | 13.0 (10.7-13.9) | | 7.2 (3.3-11.6) | |  |
| **Duration of OCP use (yrs) in those who had used it** | 10 (3-12) | | 6 (3-8) | | 5 (3-8) | | 6 (4-8) | |  |
| **Miscarriages (pregnancies < 6mths)** | 0 (0-1) | | 0 (0-0) | | 0 (0-1) | | 0 (0-0) | |  |
| **No. of children (pregnancies > 6mths)** | 2 (0-2) | | 2 (2-2) | | 2 (1-2) | | 2 (2-3) | |  |
| **Height (cms)** | 162.6 (158.8-167.6) | | 162.6 (157.5-165.1) | | 162.6 (157.5-166.4) | | 162.6 (157.5-165.1) | |  |
| **Weight (kg)** | 65.3 (62.6-74.0) | | 69.9 (62.6-78.9) | | 71.2 (66.7-79.2) | | 65.9 (61.7-75.8) | |  |
|  | **Number (%)** | | | | | | | |  |
|  | **No.** | **%** | **No.** | **%** | **No.** | **%** | **No.** | **%** |  |
| **Ethnicity:** |  |  |  |  |  |  |  |  |  |
| **White** | 30 | 96.8% | 48 | 98.0% | 18 | 94.7% | 30 | 100.0% |  |
| **Black** | 0 | 0.0% | 0 | 0.0% | 0 | 0.0% | 0 | 0.0% |  |
| **Asian** | 0 | 0.0% | 0 | 0.0% | 0 | 0.0% | 0 | 0.0% |  |
| **Other** | 1 | 3.2% | 1 | 2.0% | 1 | 5.3% | 0 | 0.0% |  |
| **Missing** | 0 | 0.0% | 0 | 0.0% | 0 | 0.0% | 0 | 0.0% |  |
| **Hysterectomy** | 1 | 3.2% | 6 | 12.2% | 2 | 10.5% | 4 | 13.3% |  |
| **Ever use of oral contraceptive pill** | 18 | 58.1% | 21 | 42.9% | 9 | 47.4% | 12 | 40.0% |  |
| **Use of HRT at recruitment** | 8 | 25.8% | 9 | 18.4% | 3 | 15.8% | 6 | 20.0% |  |
| **Personal history of cancer*** | 0 | 0.0% | 2 | 4.1% | 1 | 5.3% | 1 | 3.3% |  |
| **Personal history of breast cancer** | 0 | 0.0% | 1 | 2.0% | 1 | 5.3% | 0 | 0.0% |  |
| **Maternal history of ovarian cancer** | 0 | 0.0% | 1 | 2.0% | 0 | 0.0% | 1 | 3.3% |  |
| **Maternal history of breast cancer** | 4 | 12.9% | 4 | 8.2% | 1 | 5.3% | 3 | 10.0% |  |
| * includes those with personal history of breast cancer | | | | | |  |  |  |  |
